# (Acetato-κ*O*)(2,2′-bipyridine-κ^2^ *N*,*N*′)trimethyl­platinum(IV) monohydrate

**DOI:** 10.1107/S160053681000499X

**Published:** 2010-02-13

**Authors:** Cornelia Vetter, Christoph Wagner, Dirk Steinborn

**Affiliations:** a Anorganische Chemie, Institut für Chemie, Martin-Luther-Universität, Kurt-Mothes-Strasse 2, Halle-Wittenberg, D-06120 Halle, Germany

## Abstract

In the title hydrate, [Pt(CH_3_)_3_(CH_3_COO)(C_10_H_8_N_2_)]·H_2_O, the Pt^IV^ atom exhibits a distorted octa­hedral coordination geometry built up by three methyl ligands in a facial arrangement, a bipyridine ligand and a monodentately bound acetate ligand. In the crystal structure, inter­molecular O—H⋯O hydrogen bonds are observed between the water mol­ecule and the platinum complex, which link the mol­ecules into chains along the *c* axis.

## Related literature

For ligand-substitution reactions of platinum complexes, see: Vetter *et al.* (2006 ▶); Clegg *et al.* (1972 ▶); Lindner *et al.* (2008 ▶); Steinborn & Junicke (2000 ▶). For a description of the Cambridge Structural Database, see: Allen (2002 ▶).
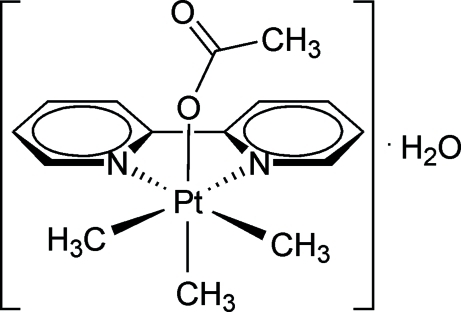


## Experimental

### 

#### Crystal data

[Pt(CH_3_)_3_(C_2_H_3_O_2_)(C_10_H_8_N_2_)]·H_2_O
*M* *_r_* = 473.44
Monoclinic, 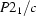

*a* = 10.972 (3) Å
*b* = 13.455 (3) Å
*c* = 13.768 (3) Å
β = 125.05 (3)°
*V* = 1663.9 (8) Å^3^
*Z* = 4
Mo *K*α radiation
μ = 8.44 mm^−1^
*T* = 293 K
0.48 × 0.34 × 0.24 mm

#### Data collection

Stoe STADI-IV diffractometer
Absorption correction: ψ scan (*X-RED32*; Stoe & Cie, 1996 ▶) *T*_min_ = 0.031, *T*_max_ = 0.089
4494 measured reflections
2931 independent reflections
2455 reflections with *I* > 2σ(*I*)
*R*_int_ = 0.031
2 standard reflections every 60 min  intensity decay: random, +−5%

#### Refinement

*R*[*F* ^2^ > 2σ(*F* ^2^)] = 0.040
*wR*(*F* ^2^) = 0.119
*S* = 1.06
2931 reflections
199 parameters
2 restraints
H atoms treated by a mixture of independent and constrained refinement
Δρ_max_ = 1.61 e Å^−3^
Δρ_min_ = −1.79 e Å^−3^

### 

Data collection: *STADI4* (Stoe & Cie, 1996 ▶); cell refinement: *STADI4*; data reduction: *STADI4*; program(s) used to solve structure: *SHELXS97* (Sheldrick, 2008 ▶); program(s) used to refine structure: *SHELXL97* (Sheldrick, 2008 ▶); molecular graphics: *DIAMOND* (Brandenburg, 2001 ▶); software used to prepare material for publication: *SHELXL97*.

## Supplementary Material

Crystal structure: contains datablocks I, global. DOI: 10.1107/S160053681000499X/tk2621sup1.cif

Structure factors: contains datablocks I. DOI: 10.1107/S160053681000499X/tk2621Isup2.hkl

Additional supplementary materials:  crystallographic information; 3D view; checkCIF report

## Figures and Tables

**Table:** 

C1—Pt1	2.036 (10)
C2—Pt1	2.041 (11)
C3—Pt1	2.032 (9)
N1—Pt1	2.161 (7)
N2—Pt1	2.152 (7)
O1—Pt1	2.168 (6)

**Table:** 

C1—Pt1—C2	85.1 (5)
C1—Pt1—N2	99.9 (4)
C2—Pt1—N2	174.8 (5)
C1—Pt1—N1	176.5 (4)
C2—Pt1—N1	98.2 (4)
N2—Pt1—N1	76.7 (3)
C3—Pt1—O1	176.2 (4)

**Table 2:** Hydrogen-bond geometry (Å, °)

*D*—H⋯*A*	*D*—H	H⋯*A*	*D*⋯*A*	*D*—H⋯*A*
O3—H22⋯O1	0.88 (11)	1.96 (11)	2.836 (12)	172 (15)
O3—H21⋯O2^i^	0.85 (9)	1.96 (10)	2.810 (14)	177 (11)

Symmetry code: (i) 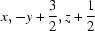.

**Fig. 1.**
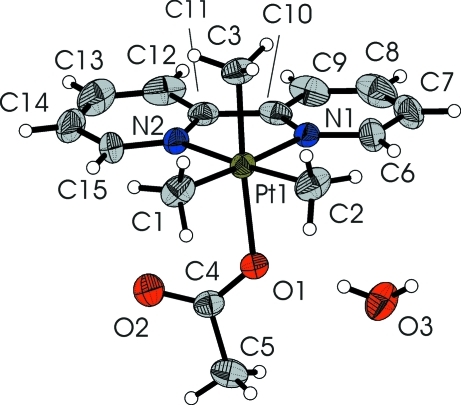
Structure of the asymmetric unit of the title hydrate [PtMe3(OAc-κO)(bpy)].H2O. Displacement ellipsoids are drawn at the 30% probability level and the H atoms are shown as small spheres of arbitrary radii.

## supplementary crystallographic information

### Comment

Due to the low-spin *d*^6^ electron configuration of platinum(IV), ligand substitution reactions of their complexes may be hampered. Starting from complexes having a PtMe_3_ unit (Vetter *et al.*, 2006; Clegg *et al.*, 1972; Lindner *et al.*, 2008), substitution reactions were found to proceed smoothly even with weak donors (Steinborn & Junicke, 2000) because the leaving ligand is additionally activated by the high *trans* effect exerted by the methyl ligand.

The asymmetric unit of the title hydrate comprises a neutral platinum complex, [PtMe_3_(OAc-κ*O*)(bpy)], and a water molecule. The primary coordination sphere of the platinum atom is built up by three methyl ligands in *facial* binding fashion, a bipyridine ligand and a monodentately bound acetato ligand. As expected for Pt(IV) complexes, an octahedral coordination geometry was found, which is distorted due to the restricted bite of the 2,2'-bipyridine ligand [N1—Pt1—N2 76.7 (3)°]; the other angles between *cis* arranged ligands are between 85.1 (5) and 99.9 (4)°. Due to the high *trans* influence of the methyl ligands the Pt1—O1 bond was found to be relatively long (2.168 (6) Å) compared to those of other carboxylato platinum(IV) [median: 2.013, lower/upper quartile: 2.001/2.044 Å, 496 observations taken from the CSD, version 5.30 (Allen, 2002)]. In the crystal structure quite strong intermolecular O—H···O hydrogen bonds were found in which the water molecules act as hydrogen donors and the oxygen atoms of acetato ligand as hydrogen acceptors (Table 1). Due to these hydrogen bonds the molecules are linked in infinite chains along the *c* axis.

### Experimental

Under anaerobic conditions [(PtMe_3_I)_4_] (50 mg, 0.03 mmol) and AgOAc (23 mg, 0.14 mmol) were stirred in acetone (10 ml) for 15 h in the absence of light. The precipitated AgI was filtered off and the solvent was reduced *in vacuo* to 3 ml. Then *n*-pentane was added and the white precipitate was collected by filtration, washed with *n*-pentane (2 × 1 ml) and recrystallized from chloroform.

### Refinement

The water-H atoms were found in a difference map and refined with each O—H distance restrained to 0.85 (1) Å. All other H atoms were positioned geometrically and allowed to ride on the respective parent atoms with C—H = 0.93–0.96 Å [*U*_iso_(H) = 1.2 *U*_eq_(C)]. The maximum and minimum residual electron density peaks of 1.61 and -1.79 e Å^-3^, respectively, were located 1.19 Å and 1.21 Å from the Pt1 atom.

### Crystal data

**Table:**

[Pt(CH_3_)_3_(C_2_H_3_O_2_)(C_10_H_8_N_2_)]·H_2_O	*F*(000) = 912
*M**_r_* = 473.44	*D*_x_ = 1.890 Mg m^−^^3^
Monoclinic, *P*2_1_/*c*	Mo *K*α radiation, λ = 0.71073 Å
Hall symbol: -P 2ybc	Cell parameters from 26 reflections
*a* = 10.972 (3) Å	θ = 15.1–25.2°
*b* = 13.455 (3) Å	µ = 8.44 mm^−^^1^
*c* = 13.768 (3) Å	*T* = 293 K
β = 125.05 (3)°	Block, orange
*V* = 1663.9 (8) Å^3^	0.48 × 0.34 × 0.24 mm
*Z* = 4	

### Data collection

**Table:**

Stoe STADI-IV diffractometer	2455 reflections with *I* > 2σ(*I*)
Radiation source: fine-focus sealed tube	*R*_int_ = 0.031
graphite	θ_max_ = 25.0°, θ_min_ = 2.3°
ω/2θ scans	*h* = −13→13
Absorption correction: ψ scan (*X-RED32*; Stoe & Cie, 1996)	*k* = −16→0
*T*_min_ = 0.031, *T*_max_ = 0.089	*l* = −13→16
4494 measured reflections	2 standard reflections every 60 min
2931 independent reflections	intensity decay: random, +−5%

### Refinement

**Table:**

Refinement on *F*^2^	Secondary atom site location: difference Fourier map
Least-squares matrix: full	Hydrogen site location: inferred from neighbouring sites
*R*[*F*^2^ > 2σ(*F*^2^)] = 0.040	H atoms treated by a mixture of independent and constrained refinement
*wR*(*F*^2^) = 0.119	*w* = 1/[σ^2^(*F*_o_^2^) + (0.0676*P*)^2^ + 4.3682*P*] where *P* = (*F*_o_^2^ + 2*F*_c_^2^)/3
*S* = 1.06	(Δ/σ)_max_ = 0.001
2931 reflections	Δρ_max_ = 1.61 e Å^−^^3^
199 parameters	Δρ_min_ = −1.79 e Å^−^^3^
2 restraints	Extinction correction: *SHELXL* (Sheldrick, 2008), Fc^*^=kFc[1+0.001xFc^2^λ^3^/sin(2θ)]^-1/4^
0 constraints	Extinction coefficient: 0.0018 (3)
Primary atom site location: structure-invariant direct methods	

### Special details

**Table:**

Geometry. All esds (except the esd in the dihedral angle between two l.s. planes) are estimated using the full covariance matrix. The cell esds are taken into account individually in the estimation of esds in distances, angles and torsion angles; correlations between esds in cell parameters are only used when they are defined by crystal symmetry. An approximate (isotropic) treatment of cell esds is used for estimating esds involving l.s. planes.
Refinement. Refinement of *F*^2^ against ALL reflections. The weighted *R*-factor wR and goodness of fit *S* are based on *F*^2^, conventional *R*-factors *R* are based on *F*, with *F* set to zero for negative *F*^2^. The threshold expression of *F*^2^ > σ(*F*^2^) is used only for calculating *R*-factors(gt) etc. and is not relevant to the choice of reflections for refinement. *R*-factors based on *F*^2^ are statistically about twice as large as those based on *F*, and *R*- factors based on ALL data will be even larger.

### Fractional atomic coordinates and isotropic or equivalent isotropic displacement parameters (Å^2^)

**Table:**

	*x*	*y*	*z*	*U*_iso_*/*U*_eq_	
C1	−0.0620 (12)	0.7592 (8)	0.1850 (9)	0.072 (3)	
H1	−0.0535	0.7673	0.1198	0.087*	
H3	−0.1629	0.7714	0.1584	0.087*	
H2	−0.0345	0.6926	0.2150	0.087*	
C2	−0.0048 (12)	0.7967 (10)	0.4045 (11)	0.081 (3)	
H6	0.0412	0.8284	0.4804	0.097*	
H5	0.0170	0.7269	0.4158	0.097*	
H4	−0.1106	0.8063	0.3591	0.097*	
C3	−0.0935 (11)	0.9574 (8)	0.2409 (10)	0.069 (3)	
H9	−0.0603	1.0175	0.2869	0.083*	
H8	−0.1760	0.9304	0.2387	0.083*	
H7	−0.1241	0.9718	0.1616	0.083*	
C4	0.3091 (10)	0.7018 (7)	0.3608 (9)	0.058 (2)	
C5	0.4295 (13)	0.6271 (9)	0.4412 (12)	0.081 (4)	
H11	0.4636	0.5950	0.3986	0.098*	
H10	0.3899	0.5782	0.4667	0.098*	
H12	0.5113	0.6608	0.5091	0.098*	
C6	0.2491 (12)	0.9787 (10)	0.5540 (9)	0.077 (3)	
H13	0.2051	0.9358	0.5784	0.093*	
C7	0.3426 (14)	1.0548 (11)	0.6304 (10)	0.092 (4)	
H14	0.3583	1.0638	0.7038	0.110*	
C8	0.4096 (15)	1.1153 (11)	0.5953 (15)	0.098 (5)	
H15	0.4738	1.1652	0.6454	0.117*	
C9	0.3823 (12)	1.1025 (9)	0.4859 (13)	0.085 (4)	
H16	0.4276	1.1437	0.4611	0.102*	
C10	0.2869 (9)	1.0279 (7)	0.4123 (9)	0.058 (2)	
C11	0.2542 (10)	1.0116 (7)	0.2937 (9)	0.057 (2)	
C12	0.3080 (12)	1.0709 (9)	0.2434 (14)	0.085 (4)	
H17	0.3648	1.1271	0.2829	0.102*	
C13	0.2752 (16)	1.0446 (13)	0.1327 (15)	0.098 (5)	
H18	0.3103	1.0832	0.0975	0.117*	
C14	0.1935 (17)	0.9642 (12)	0.0776 (12)	0.091 (4)	
H19	0.1719	0.9459	0.0041	0.109*	
C15	0.1410 (13)	0.9078 (9)	0.1301 (9)	0.069 (3)	
H20	0.0846	0.8514	0.0911	0.083*	
N1	0.2221 (8)	0.9665 (5)	0.4471 (6)	0.0486 (16)	
N2	0.1687 (8)	0.9318 (6)	0.2344 (6)	0.0525 (17)	
O1	0.2551 (7)	0.7497 (5)	0.4062 (6)	0.0615 (17)	
O2	0.2727 (10)	0.7105 (7)	0.2563 (8)	0.089 (2)	
O3	0.4338 (10)	0.7587 (8)	0.6577 (9)	0.088 (3)	
H21	0.382 (12)	0.767 (9)	0.685 (10)	0.07 (4)*	
H22	0.379 (15)	0.763 (12)	0.580 (10)	0.12 (6)*	
Pt1	0.07572 (3)	0.85719 (3)	0.31606 (3)	0.04758 (19)	

### Atomic displacement parameters (Å^2^)

**Table:**

	*U*^11^	*U*^22^	*U*^33^	*U*^12^	*U*^13^	*U*^23^
C1	0.077 (7)	0.052 (6)	0.069 (7)	−0.020 (5)	0.031 (6)	0.003 (5)
C2	0.070 (6)	0.095 (9)	0.094 (8)	0.007 (6)	0.057 (6)	0.031 (7)
C3	0.057 (5)	0.067 (7)	0.075 (7)	0.015 (5)	0.033 (5)	0.015 (5)
C4	0.058 (5)	0.050 (5)	0.066 (6)	0.001 (4)	0.036 (5)	0.008 (5)
C5	0.071 (7)	0.076 (8)	0.100 (9)	0.017 (6)	0.050 (7)	0.004 (6)
C6	0.075 (7)	0.102 (9)	0.054 (6)	0.018 (6)	0.036 (5)	−0.005 (6)
C7	0.079 (8)	0.108 (11)	0.054 (6)	0.023 (8)	0.018 (6)	−0.027 (7)
C8	0.069 (8)	0.082 (9)	0.107 (12)	−0.003 (6)	0.030 (8)	−0.035 (8)
C9	0.055 (6)	0.068 (7)	0.103 (10)	−0.002 (5)	0.029 (6)	−0.020 (7)
C10	0.043 (4)	0.049 (5)	0.073 (6)	0.009 (4)	0.027 (4)	−0.003 (4)
C11	0.055 (5)	0.055 (6)	0.072 (6)	0.018 (4)	0.042 (5)	0.017 (5)
C12	0.066 (6)	0.070 (7)	0.134 (12)	0.007 (6)	0.067 (7)	0.031 (8)
C13	0.095 (9)	0.120 (13)	0.116 (12)	0.016 (8)	0.083 (9)	0.047 (10)
C14	0.106 (9)	0.121 (12)	0.079 (8)	0.039 (9)	0.072 (8)	0.037 (8)
C15	0.088 (7)	0.078 (7)	0.058 (6)	0.014 (6)	0.051 (6)	0.003 (5)
N1	0.048 (4)	0.051 (4)	0.048 (4)	0.009 (3)	0.029 (3)	0.006 (3)
N2	0.056 (4)	0.058 (4)	0.052 (4)	0.008 (4)	0.036 (3)	0.009 (4)
O1	0.063 (4)	0.062 (4)	0.055 (4)	0.014 (3)	0.032 (3)	0.006 (3)
O2	0.108 (6)	0.095 (6)	0.084 (6)	0.025 (5)	0.067 (5)	0.014 (5)
O3	0.070 (5)	0.118 (8)	0.068 (5)	−0.001 (5)	0.035 (5)	0.014 (5)
Pt1	0.0491 (2)	0.0487 (3)	0.0473 (3)	0.00074 (14)	0.02902 (18)	0.00514 (14)

### Geometric parameters (Å, °)

**Table:**

C1—Pt1	2.036 (10)	C7—H14	0.9300
C1—H1	0.9600	C8—C9	1.37 (2)
C1—H3	0.9600	C8—H15	0.9300
C1—H2	0.9600	C9—C10	1.382 (15)
C2—Pt1	2.041 (11)	C9—H16	0.9300
C2—H6	0.9600	C10—N1	1.345 (13)
C2—H5	0.9600	C10—C11	1.474 (15)
C2—H4	0.9600	C11—N2	1.349 (13)
C3—Pt1	2.032 (9)	C11—C12	1.391 (15)
C3—H9	0.9600	C12—C13	1.40 (2)
C3—H8	0.9600	C12—H17	0.9300
C3—H7	0.9600	C13—C14	1.33 (2)
C4—O2	1.258 (13)	C13—H18	0.9300
C4—O1	1.259 (12)	C14—C15	1.381 (17)
C4—C5	1.518 (14)	C14—H19	0.9300
C5—H11	0.9600	C15—N2	1.326 (12)
C5—H10	0.9600	C15—H20	0.9300
C5—H12	0.9600	N1—Pt1	2.161 (7)
C6—N1	1.335 (13)	N2—Pt1	2.152 (7)
C6—C7	1.403 (18)	O1—Pt1	2.168 (6)
C6—H13	0.9300	O3—H21	0.85 (9)
C7—C8	1.36 (2)	O3—H22	0.88 (11)
			
Pt1—C1—H1	109.5	N1—C10—C11	117.4 (8)
Pt1—C1—H3	109.5	C9—C10—C11	121.4 (11)
H1—C1—H3	109.5	N2—C11—C12	120.2 (11)
Pt1—C1—H2	109.5	N2—C11—C10	115.6 (8)
H1—C1—H2	109.5	C12—C11—C10	124.2 (11)
H3—C1—H2	109.5	C11—C12—C13	118.7 (13)
Pt1—C2—H6	109.5	C11—C12—H17	120.6
Pt1—C2—H5	109.5	C13—C12—H17	120.6
H6—C2—H5	109.5	C14—C13—C12	119.7 (12)
Pt1—C2—H4	109.5	C14—C13—H18	120.2
H6—C2—H4	109.5	C12—C13—H18	120.2
H5—C2—H4	109.5	C13—C14—C15	119.7 (13)
Pt1—C3—H9	109.5	C13—C14—H19	120.1
Pt1—C3—H8	109.5	C15—C14—H19	120.1
H9—C3—H8	109.5	N2—C15—C14	121.9 (12)
Pt1—C3—H7	109.5	N2—C15—H20	119.1
H9—C3—H7	109.5	C14—C15—H20	119.1
H8—C3—H7	109.5	C6—N1—C10	119.3 (9)
O2—C4—O1	126.2 (9)	C6—N1—Pt1	126.3 (8)
O2—C4—C5	117.9 (10)	C10—N1—Pt1	114.4 (6)
O1—C4—C5	116.0 (10)	C15—N2—C11	119.8 (9)
C4—C5—H11	109.5	C15—N2—Pt1	124.8 (8)
C4—C5—H10	109.5	C11—N2—Pt1	115.4 (6)
H11—C5—H10	109.5	C4—O1—Pt1	126.0 (6)
C4—C5—H12	109.5	H21—O3—H22	112 (10)
H11—C5—H12	109.5	C3—Pt1—C1	89.0 (5)
H10—C5—H12	109.5	C3—Pt1—C2	89.2 (5)
N1—C6—C7	121.4 (13)	C1—Pt1—C2	85.1 (5)
N1—C6—H13	119.3	C3—Pt1—N2	89.6 (4)
C7—C6—H13	119.3	C1—Pt1—N2	99.9 (4)
C8—C7—C6	118.8 (13)	C2—Pt1—N2	174.8 (5)
C8—C7—H14	120.6	C3—Pt1—N1	89.8 (4)
C6—C7—H14	120.6	C1—Pt1—N1	176.5 (4)
C7—C8—C9	119.7 (13)	C2—Pt1—N1	98.2 (4)
C7—C8—H15	120.1	N2—Pt1—N1	76.7 (3)
C9—C8—H15	120.1	C3—Pt1—O1	176.2 (4)
C8—C9—C10	119.6 (14)	C1—Pt1—O1	92.5 (4)
C8—C9—H16	120.2	C2—Pt1—O1	87.4 (4)
C10—C9—H16	120.2	N2—Pt1—O1	93.6 (3)
N1—C10—C9	121.2 (11)	N1—Pt1—O1	88.9 (3)
			
N1—C6—C7—C8	−1.9 (18)	C12—C11—N2—Pt1	−173.8 (7)
C6—C7—C8—C9	2(2)	C10—C11—N2—Pt1	7.6 (9)
C7—C8—C9—C10	−0.1 (19)	O2—C4—O1—Pt1	3.0 (15)
C8—C9—C10—N1	−1.1 (16)	C5—C4—O1—Pt1	−177.2 (7)
C8—C9—C10—C11	179.9 (10)	C15—N2—Pt1—C3	−92.8 (8)
N1—C10—C11—N2	−4.0 (12)	C11—N2—Pt1—C3	83.5 (7)
C9—C10—C11—N2	175.0 (9)	C15—N2—Pt1—C1	−3.8 (9)
N1—C10—C11—C12	177.5 (9)	C11—N2—Pt1—C1	172.4 (6)
C9—C10—C11—C12	−3.5 (14)	C15—N2—Pt1—N1	177.3 (8)
N2—C11—C12—C13	−1.7 (15)	C11—N2—Pt1—N1	−6.4 (6)
C10—C11—C12—C13	176.7 (10)	C15—N2—Pt1—O1	89.3 (8)
C11—C12—C13—C14	0.2 (19)	C11—N2—Pt1—O1	−94.5 (6)
C12—C13—C14—C15	0(2)	C6—N1—Pt1—C3	93.0 (9)
C13—C14—C15—N2	0.5 (18)	C10—N1—Pt1—C3	−85.4 (7)
C7—C6—N1—C10	0.7 (15)	C6—N1—Pt1—C2	3.8 (9)
C7—C6—N1—Pt1	−177.7 (8)	C10—N1—Pt1—C2	−174.6 (6)
C9—C10—N1—C6	0.8 (13)	C6—N1—Pt1—N2	−177.4 (8)
C11—C10—N1—C6	179.8 (8)	C10—N1—Pt1—N2	4.2 (6)
C9—C10—N1—Pt1	179.3 (7)	C6—N1—Pt1—O1	−83.4 (8)
C11—C10—N1—Pt1	−1.7 (10)	C10—N1—Pt1—O1	98.2 (6)
C14—C15—N2—C11	−2.0 (15)	C4—O1—Pt1—C1	53.9 (8)
C14—C15—N2—Pt1	174.0 (8)	C4—O1—Pt1—C2	138.9 (9)
C12—C11—N2—C15	2.6 (13)	C4—O1—Pt1—N2	−46.2 (8)
C10—C11—N2—C15	−176.0 (8)	C4—O1—Pt1—N1	−122.8 (8)

### Hydrogen-bond geometry (Å, °)

**Table:**

*D*—H···*A*	*D*—H	H···*A*	*D*···*A*	*D*—H···*A*
O3—H22···O1	0.88 (11)	1.96 (11)	2.836 (12)	172 (15)
O3—H21···O2^i^	0.85 (9)	1.96 (10)	2.810 (14)	177 (11)

Symmetry codes: (i) *x*, −*y*+3/2, *z*+1/2.
